# Characteristics of Non-linguistic Cognitive Impairment in Post-stroke Aphasia Patients

**DOI:** 10.3389/fneur.2020.01038

**Published:** 2020-09-30

**Authors:** Jingfan Yao, Xinxin Liu, Qi Liu, Jinfang Wang, Na Ye, Xiao Lu, Yishuang Zhao, Hongyan Chen, Zaizhu Han, Miaoxin Yu, Yu Wang, Gaifen Liu, Yumei Zhang

**Affiliations:** ^1^Department of Neurology, Beijing Tiantan Hospital, Capital Medical University, Beijing, China; ^2^China National Clinical Research Center for Neurological Diseases, Beijing, China; ^3^Beijing Office for Cerebrovascular Disease Prevention and Control, Beijing, China; ^4^Department of Neurology, Tianjin First Central Hospital, Tianjin, China; ^5^Department of Neurology, General Hospital of the Yang Tze River Shipping, Wuhan Brain Hospital, Wuhan, China; ^6^Department of Rehabilitation Medicine, Beijing Tiantan Hospital, Capital Medical University, Beijing, China; ^7^Department of Neuroradiology, Beijing Tiantan Hospital, Capital Medical University, Beijing, China; ^8^State Key Laboratory for Cognitive Neuroscience and Learning, Beijing Normal University, Beijing, China

**Keywords:** post-stroke aphasia, non-linguistic cognitive impairment, LOTCA, fluent aphasia, non-fluent aphasia

## Abstract

**Background:** Non-linguistic cognitive training has been suggested to improve the communication skills of patients with post-stroke aphasia (PSA). However, the association between language and non-linguistic cognitive functions is not fully understood. In this study, we used the Loewenstein Occupational Therapy Cognitive Assessment (LOTCA) to evaluate the characteristics of non-linguistic cognitive impairments in Chinese PSA patients.

**Methods:** A total of 86 stroke patients were recruited in this study. Language and non-linguistic cognitive impairments were evaluated by the Western Aphasia Battery (WAB) and LOTCA, respectively. The patients were divided into two groups (PSA and non-PSA), and the Chinese norm (the data came from 44 Chinese individuals without neurological disorders in a previous study) was used as the control group. The LOTCA scores were compared among the three groups. Patients in the PSA group were subdivided into the fluent aphasia group (FAG) and the non-FAG according to the Chinese aphasia fluency characteristic scale. The LOTCA scores were also compared between the PSA subdivisions. Potential confounders were adjusted in the analysis of covariance. Partial correlation analysis between the subscores of WAB and LOTCA was also performed.

**Results:** The total LOTCA scores in the PSA group (75.11 ± 17.08) were significantly lower compared with scores in the non-PSA (96.80 ± 7.75, *P* < 0.001) and the control group (97.65 ± 16.24, *P* < 0.001). The PSA group also had lower orientation, visual perception (VP), spatial perception (SP), visuomotor organization, thinking operation, and attention scores. The total LOTCA, orientation, VP, SP, and MP scores were lower in the non-FAG (69.24 ± 18.06, 8.62 ± 5.09, 12.76 ± 2.47, 7.48 ± 3.01, and 9.62 ± 2.25, respectively) compared with the FAG (80.36 ± 14.07, 12.14 ± 3.99, 14.09 ± 1.93, 9.68 ± 3.01, 10.55 ± 1.63, respectively, *P*'s < 0.05). The aphasia quotient was positively correlated with the total score of LOTCA and scores of orientation, VP, SP, and MP (*r* = 0.710, 0.744, 0.565, 0.597, and 0.616; *P* < 0.001).

**Conclusion:** Compared with stroke patients without aphasia, patients with PSA often have more extensive and serious non-linguistic cognitive impairments. Patients with non-fluent aphasia often present with serious cognitive impairments than those with fluent aphasia, especially the impairments of orientation and SP. Non-linguistic cognitive impairments correlate with language impairments in aphasia.

## Introduction

Aphasia occurs in about 30% of stroke patients and is characterized by impairments in oral fluency, comprehension, repetition, naming, reading, or writing (1). The processing of language is the core of cognition (2) and requires the participation of other non-linguistic cognitive functions (2–5). Moreover, the overlapping neural networks of language and non-linguistic cognition (6) suggested that the language and non-linguistic cognitive function of patients with post-stroke aphasia (PSA) (3) cannot be separated.

There is growing consensus that aphasia might be accompanied with deficits of other cognitive functions (2, 7–9), such as executive function (4, 6, 10), attention (6, 11–13), visuospatial perception (14, 15), logical thinking (10), and memory (14, 16, 17), after a left hemispheric stroke. Non-linguistic cognitive impairments are associated with semantic cognitive deficits (18), comprehension difficulty (5), and other language impairments (4), which also play an important role in aphasia recovery and rehabilitation (19–25).

However, there is still challenge on the evaluation of non-linguistic cognition in aphasia. Because of the interaction between language and cognitive function, it is important to choose the cognitive tests that are independent of language skills. A behavioral study has found the significant correlation between scores of Western Aphasia Battery (WAB) and Mini Mental State Examination (MMSE), and thought MMSE could be used as a rapid screening tool for cognitive function (except for visuospatial function) in PSA patients (26). Some previous studies have focused on domain-specific tests to evaluate different cognitive functions, such as studying the impact of executive impairment on semantic cognition (18), comprehension, and naming (27), investigating the relationship between attention and language performance (12, 28). Some other studies have combined different domain-specific tests (10, 29) or chosen the comprehensive cognitive scales, such as Aphasia Check List (30), Cognitive Linguistic Quick Test (9), and Oxford Cognitive Screen (31). In recent years, researchers have begun to focus on the relationship between the images and the benefits they provide for patients with aphasia, which is essential to advance therapeutic practices (32). Thus, visual-supporting materials may be the most important assessment tools for aphasia. The Loewenstein Occupational Therapy Cognitive Assessment (LOTCA) is one kind of comprehensive scale (33, 34), which is developed by the Loewenstein Rehabilitation Hospital and widely used to evaluate the cognitive function of various brain diseases. It has the superiority of avoiding the influence of language-related factors on test results because of its use of the pictures. The Chinese version of LOTCA has been revised and verified (35), but it still needs to be further verified via larger-scale Chinese PSA patients.

This study aims to evaluate the cognitive performance of PSA by LOTCA and to compare the cognitive performance between PSA and stroke without aphasia, furtherly to investigate the relationship between non-linguistic cognitive dysfunction and language impairment.

## Materials and Methods

### Subjects

A total of 86 stroke patients from Beijing Tiantan Hospital, Capital Medical University, were recruited between May 2017 and January 2019. WAB (36) was used to diagnose aphasia. The patients were divided into the PSA and the non-PSA (stroke patients without aphasia) groups. The inclusion criteria for the PSA group were as follows: (a) native Chinese speaker, (b) able to complete all neuropsychological assessments, (c) aphasia quotient (AQ) of WAB <93.8, (d) first stroke with lesions located in the left brain hemisphere, (e) right handedness, and (f) signed informed consent. The inclusion criteria for the non-PSA group were the same as those for the PSA group except for AQ ≥98.4. The exclusion criteria for both the PSA and the non-PSA groups were as follows: (a) AQ ranging from 93.8 to 98.4 (defined as diffuse brain injury or subcortical injury); (b) existing language and cognitive impairments before stroke or combined with other neurological diseases (assessed by medical history, examination, or psychological assessment); (c) recurrent stroke, cerebellar stroke, or brainstem stroke; (d) severe dysarthria; (e) history of mental illness or depression indicated by the score of Stroke Aphasic Depression Questionnaire in Hospital, 10th Version (SADQ-H10) (Appendix 1 in Supplementary Material) (37) >6; (f) severe internal medicine diseases, hearing or vision disorders; and (g) cerebral small vessel disease, which were detectable by image markers (white matter lesions (Fazekas score >0) (38), microbleeds, lacuna, and dilated perivascular spaces) via routine brain magnetic resonance imaging (MRI) scan [including T1-weighted image, T2-weighted image, fluid-attenuated inversion recovery, diffusion-weighted imaging (DWI), apparent diffusion coefficient, and susceptibility-weighted imaging (SWI)].

In this study, the norm of the LOTCA scores was used as the control group. The norm of the LOTCA scores was obtained from 44 Chinese individuals without neurological disorders, whose average age was 55.4 ± 23.7 years, and its reliability and validity of the LOTCA (Chinese version) were verified by Yan et al. (35).

This study was approved by the Ethics Committee of the Beijing Tiantan Hospital, Capital Medical University (ethical approval no. KYSB2016-023). Signed written informed consent was given by all participants or their legal representative.

### General Clinical Information

Age, gender, educational level, days after stroke onset, medical history, and the score on the National Institutes of Health Stoke Scale (NIHSS) (to assess neurological deficits of stroke) were extracted from medical records by trained research coordinators.

### Information of Lesions

All patients underwent brain MRI or computed tomography (CT); two radiologists who were blinded to the neuropsychological assessment interpreted the images independently. Cerebral infarction was determined by DWI, and hemorrhagic lesions were determined by CT or SWI. Slicer 4.10.2 software was used to manually draw and extract lesions. Then MATLAB script was used to obtain the lesion volume (milliliter) based on the product of the number of voxels and the size of voxels in the mask.

### Neuropsychological Assessment

All patients underwent neuropsychological assessment, performed by a trained neurologist. The SADQ-H10 (Chinese version) (37) was used to exclude cognitive impairment caused by depression. The WAB was used to diagnose aphasia and to calculate the AQ and scores for spontaneous speech (information and fluency), comprehension, repetition, and naming. The scores for spontaneous speech ranged from 0 to 20. The scores for comprehension, repetition, and naming ranged from 0 to 10: AQ = (spontaneous speech + comprehension + repetition + naming) × 2, which ranged from 0 to 100. The fluency of language was judged by the Chinese aphasia fluency characteristic scale (39) (attached to Appendix 3 in Supplementary Material). This scale included parameters such as vocabulary, intonation, pronunciation, length of phrase, laborsome speech, press of speech, substantive words, grammar, and paraphasia. There were three options for each item with scores ranging from 1 to 3. The total scores ranged from a minimum of 9 to a maximum of 27. Fluent aphasia (from 21 to 27), intermediate aphasia (from 14 to 20), and non-fluent aphasia (from 9 to 13) were subsequently distinguished. Finally, the LOTCA (Chinese version) (Appendix 2 in Supplementary Material) (35) was used to evaluate the non-linguistic cognitive function of all participants. The LOTCA evaluated orientation, visuospatial perception, abstract thinking, organizational reasoning, executive function, and attention. All patients finished the following tasks with verbal and written instructions, pictures, and objects: (a) orientation included orientation to place and orientation to time; the scores ranged from 2 to 16; (b) visual perception (VP) included object identification, shape identification, overlapping figures, and object constancy; the total scores ranged from 4 to 16; and (c) spatial perception (SP) included directions on the client's body, spatial relations, and spatial relations in pictures; the total scores ranged from 3 to 12; (d) motor praxis (MP) included motor imitation, utilization of objects, and symbolic actions; the total scores ranged from 3 to 12; (e) visuomotor organization (VMO) checked the integration of perceptive movement and space; it included copying geometric forms, reproduction of two-dimensional models, pegboard construction, colored block designing, plain block designing, reproduction of a puzzle, and drawing of a clock; the total scores ranged from 7 to 28; (f) thinking operations (TOs) included pictorial classification, Riska unstructured object classification, Riska structured object classification, pictorial sequencing A and B, geometrical sequencing, and logical questions; the total scores ranged from 7 to 31; (g) attention: the scores ranged from 1 to 4; the total scores ranged from 26 to 115, and the score for attention was calculated separately. A lower score represented serious cognitive impairments.

### Statistical Analysis

Statistical Package for the Social Sciences version 25.0, SAS version 9.4 statistical software (SAS Institute Inc., Cary, NC), and PRISM 8.0 software were used to analyze the data. Continuous variables with normal distribution are presented as mean (standard deviation [SD]) and compared using *t*-test. Non-normal distribution variables were presented as median (interquartile range) and compared using the non-parametric rank sum test. Categorical variables were presented as number and percentage and were compared using the χ^2^-test or Fisher exact test. The total score and subscores of LOTCA between the PSA, non-PSA groups, and Chinese norm were compared using single analysis of variance with group as a factor and followed up with *post-hoc* tests. When comparing the total score and subscores of LOTCA between the PSA and non-PSA groups, as well as between FAG and non-FAG groups, covariance analysis was used, and the lesion volume was taken as covariate. Partial correlation analyses were performed between WAB scores and LOTCA scores in the PSA group. A two-sided *P* < 0.05 was considered to be statistically significant.

## Results

### General Clinical Characteristics

A total of 86 stroke patients were enrolled in the study. There were 45 (52.3%) PSA patients and 41 (47.7%) non-PSA patients. The general clinical characteristics are presented in Table 1. No significant differences were found on age, gender, educational level, days after stroke onset, and NIHSS scores between the PSA and the non-PSA groups (*P* = 0.52, 0.42, 0.40, 0.78, and 0.99, respectively). The volume of lesion of non-PSA group was significantly lower than that of the PSA group (*P* < 0.001).

**Table 1:** General clinical characteristics.

**Variables**	**PSA group (*n* = 45)**	**Non-PSA group (*n* = 41)**	***P-value***
Age, years ± (SD)	57.71 ± 10.77	55.39 ± 11.67	0.52
Education, years ± (SD)	12.24 ± 3.64	11.24 ± 3.24	0.40
Days after onset, days ± (SD)	24.53 ± 14.87	25.27 ± 9.29	0.78
NIHSS score	4.18 ± 2.44	3.59 ± 2.45	0.99
Male, (*n*, %)	35 (77.8)	35 (85.3)	0.42
Volume of lesion (ml)	29.62 ± 27.07	10.77 ± 10.25	<0.001

*PSA, post-stroke aphasia; NIHSS, National Institute of Health Stroke Scale; SD, standard deviation; n, number*.

### WAB and LOTCA Assessments of Subjects

The total LOTCA scores of the PSA group (75.11 ± 17.08) were significantly lower than the Chinese norm (97.65 ± 16.24; *P* < 0.001) (Table 2). The scores for orientation, VP, SP, MP, VMO, TO, and attention were all significantly lower in the PSA group than that in the Chinese norm (*P* < 0.017).

**Table 2:** Comparison of LOTCA scores among PSA group, non-PSA group, and Chinese norm.

**Variables**	**PSA group**	**Non-PSA group**	**Chinese norm**	P^*^	P^**^	P***
Total score	75.11 ± 17.08	96.80 ± 7.75	97.65 ± 16.24	<0.001	<0.001	0.787
Orientation	10.49 ± 4.82	15.17 ± 1.34	14.84 ± 2.48	<0.001	<0.001	0.642
VP	13.44 ± 2.29	15.34 ± 0.99	14.42 ± 1.48	<0.001	0.007	0.014
SP	8.60 ± 3.19	11.37 ± 0.97	10.44 ± 2.17	<0.001	<0.001	0.068
MP	10.09 ± 1.99	10.78 ± 1.41	11.42 ± 0.88	0.035	<0.001	0.052
VMO	18.13 ± 4.78	22.54 ± 3.26	22.02 ± 5.79	<0.001	<0.001	0.615
TO	14.36 ± 5.35	21.61 ± 3.84	20.56 ± 6.15	<0.001	<0.001	0.357
Attention	2.84 ± 0.74	3.32 ± 0.69	3.95 ± 0.21	<0.001	<0.001	<0.001

P*, comparison between PSA group and non-PSA group; P^**^, comparison between PSA group and Chinese norm; P^***^, comparison between non-PSA group and Chinese norm. LOTCA, Loewenstein Occupational Therapy Cognitive Assessment; VP, visual perception; SP, spatial perception; MP, motor praxis; VMO, visuomotor organization; TO, thinking operations; PSA, post-stroke aphasia.

No statistical significance was found in the total LOTCA scores between the non-PSA group and the Chinese norm (*P* = 0.787). However, a significant reduction was observed in the VP and attention scores in the non-PSA group (*P* = 0.014 and *P* < 0.001, respectively).

The total LOTCA score in the PSA group was also significantly lower than that in the non-PSA group (96.80 ± 7.75; *P* < 0.001). Significant differences in orientation, VP, SP, VMO, TO, and attention scores were observed between the PSA and the non-PSA groups (*P* < 0.001) (Table 2). PSA patients have more severe and extensive non-linguistic cognitive impairments.

Considering the lesion volume as covariate, the analysis of covariance (Table 3) showed there is no significant effect of lesion volume on the difference of LOTCA scores (except for MP) between PSA group and non-PSA group (*P* > 0.05).

**Table 3:** The effect of lesion volume on the difference of LOTCA scores between PSA group and non-PSA group.

**Dependent variables**	**Group**		**Lesion volume**	
	***F***	***P***	***F***	***P***
Total score	39.241	<0.001	2.494	0.118
Orientation	24.031	<0.001	2.380	0.127
VP	18.470	<0.001	0.102	0.750
SP	16.875	<0.001	3.765	0.056
MP	0.288	0.593	8.046	0.006
VMO	17.959	<0.001	0.307	0.581
TO	38.113	<0.001	0.447	0.506
Attention	6.572	0.012	0.378	0.541

*VP, visual perception; SP, spatial perception; MP, motor praxis; VMO, visuomotor organization; TO, thinking operations; PSA, post-stroke aphasia; LOTCA, Loewenstein Occupational Therapy Cognitive Assessment*.

**Figure 1:**
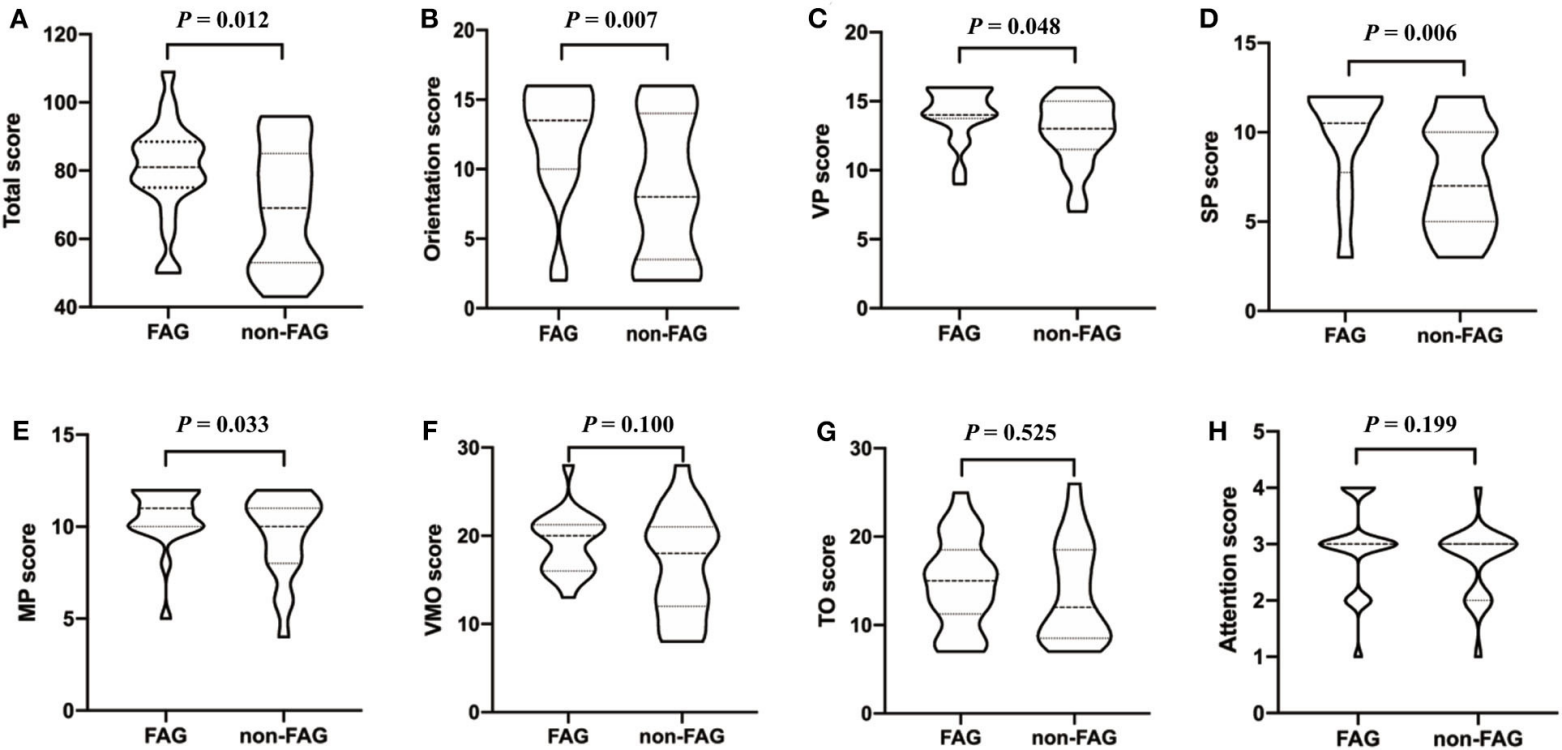
The covariance analysis of LOTCA scores between FAG group and non-FAG group. FAG, fluent aphasia group; VP, visual perception; SP, spatial perception; MP, motor praxis; VMO, visuomotor organization; TO, thinking operations; LOTCA, Loewenstein Occupational Therapy Cognitive Assessment.

The PSA group was further divided into the fluent aphasia group (FAG, *n* = 22) and the non-fluent aphasia group (non-FAG, *n* = 21) according to their oral fluency. AQ for FAG ranged from 13.7 to 92.6 with a mean of 73.99 (SD = 16.52), and AQ for non-FAG ranged from 1.5 to 87.6 with a mean of 42.29 (SD = 22.56). Two aphasia patients were judged as intermediate aphasia and thus were not included in the analysis comparing the clinical characteristics between the FAG and non-FAG groups. There was no statistically significant difference in the general clinical characteristics on gender, educational level, stroke type, days after onset, and lesion volume between the FAG and non-FAG groups (Table 4). However, statistically significant difference in the age and NIHSS score (*P* = 0.047 and *P* = 0.009, respectively) was found between these two groups (Table 4). Furthermore, the WAB scores were significantly lower in non-fluent aphasia patients compared to patients with fluent aphasia (*P* < 0.05). The total LOTCA score and scores of orientation, VP, SP, and MP in non-FAG patients were 69.24 ± 18.06, 8.62 ± 5.09, 12.76 ± 2.47, 7.48 ± 3.01, and 9.62 ± 2.25, respectively, which were significantly lower than that in FAG patients (80.36 ± 14.07, *P* = 0.012; 12.14 ± 3.99, *P* = 0.007; 14.09 ± 1.93, *P* = 0.048; 9.68 ± 3.01, *P* = 0.006; 10.55 ± 1.63, *P* = 0.033, respectively) (Figure 1).

**Table 4:** Comparison of general characteristics and WAB or LOTCA scores between fluent aphasia and non-fluent aphasia patients.

**Variables**	**FAG (*n* = 22)**	**Non-FAG (*n* = 21)**	***P***
**General clinical information**			
Age, years ± SD	61.09 ± 7.78	54.67 ± 12.40	0.047
Male, *n* (%)	16 (72.7)	17 (81.0)	0.52
Education, years ± SD	11.41 ± 3.73	13.00 ± 3.59	0.16
Stroke type			0.96
CI	20 (90.9)	19 (90.5)	
ICH	2 (9.1)	2 (9.5)	
Days after onset, days ± SD	25.41 ± 17.00	23.05 ± 12.93	0.61
NIHSS score	3.27 ± 2.05	5.14 ± 2.44	0.009
Volume of lesion, mL	34.34 ± 27.56	24.67 ± 26.30	0.45
**WAB**			
AQ	73.99 ± 16.52	42.29 ± 22.56	<0.001
Spontaneous speech	14.23 ± 2.89	7.43 ± 4.85	<0.001
Comprehension	7.64 ± 2.17	5.95 ± 2.73	0.03
Repetition	8.38 ± 2.35	4.53 ± 3.56	<0.001
Naming	6.75 ± 2.33	3.23 ± 2.98	<0.001

*FAG, fluent aphasia group; CI, cerebral infarction; ICH, intracranial hemorrhage; NIHSS, National Institute of Health Stroke Scale; WAB, western aphasia battery; AQ, aphasia quotient; n, number*.

The analysis of covariance showed there is no significant effect of lesion volume on the difference of LOTCA scores (except for SP score, *P* = 0.044) between the FAG group and non-FAG group (*P* > 0.05) (Table 5). We also included the NIHSS score and age into the analysis of covariance and found that they had no significant effect on the difference in LOTCA score.

**Table 5:** The effect of lesion volume on the difference of LOTCA scores between FAG group and non-FAG group.

**Dependent variables**	**Group**		**Lesion volume**	
	***F***	***P***	***F***	***P***
Total score	6.562	0.014	2.658	0.111
Orientation	8.001	0.007	2.644	0.112
VP	4.082	0.050	0.281	0.599
SP	8.012	0.007	4.326	0.044
MP	4.650	0.037	7.809	0.008
VMO	2.738	0.106	0.430	0.516
TO	0.379	0.542	0.327	0.571
Attention	1.419	0.241	0.458	0.502

*VP, visual perception; SP, spatial perception; MP, motor praxis; VMO, visuomotor organization; TO, thinking operations; FAG, fluent aphasia group; LOTCA, Loewenstein Occupational Therapy Cognitive Assessment*.

### Relationship Between WAB Scores and LOTCA Scores of PSA Patients

To investigate the interaction of non-linguistic cognitive impairment and language function in PSA patients, the relationship between LOTCA scores and WAB scores was analyzed. Age, gender, days after stroke onset, educational years, lesion volume, and NIHSS scores were used as covariates for partial correlation analysis. The Bonferroni correction for *P*-value was performed according to the multiple comparisons with the adjusted *P* = 0.05/40 = 0.00125. The AQ positively correlated with the total LOTCA scores (*r* = 0.710, *P* < 0.001). This showed an indication that the severity of aphasia also positively correlated with the severity of non-linguistic cognitive impairment (Table 6). There were also positive correlations between AQ and the orientation, VP, SP, and MP scores, with the exception of VMO, TO, and attention. The scores for spontaneous speech positively correlated with the scores for orientation, SP, MP, and TO (*r* = 0.613, *P* < 0.001; *r* = 0.526, *P* = 0.001; *r* = 0.519, *P* = 0.001; and *r* = 0.480, *P* = 0.001, respectively). The scores for comprehension also positively correlated with the scores for orientation, SP, and MP (*r* = 0.597, *P* < 0.001; *r* = 0.664, *P* < 0.001; and *r* = 0.607, *P* < 0.001, respectively). The scores for repetition positively correlated with the scores for orientation, MP, and VMO (*r* = 0.745, *P* < 0.001; *r* = 0.589, *P* < 0.001; and *r* = 0.518, *P* = 0.001, respectively). The score for naming positively correlated with the scores for orientation and SP (*r* = 0.594, *P* < 0.001; *r* = 0.576, *P* < 0.001, respectively). Thus, almost all the subscores of LOTCA correlated with AQ with the exception of attention, which did not correlate with all the WAB subscores. These results showed the close relationship between language function and non-linguistic cognitive function in PSA patients.

**Table 6:** Partial correlation analysis between LOTCA scores and WAB scores of the PSA group.

		**Spontaneous speech**	**Comprehension**	**Repetition**	**Naming**	**AQ**
Orientation	*R*	0.613	0.597	0.745	0.594	0.744
	*P*	<0.001	<0.001	<0.001	<0.001	<0.001
VP	*R*	0.486	0.495	0.523	0.428	0.565
	*P*	0.002	0.002	0.001	0.008	<0.001
SP	*R*	0.526	0.664	0.317	0.576	0.597
	*P*	0.001	<0.001	0.056	<0.001	<0.001
MP	*R*	0.519	0.607	0.589	0.414	0.616
	*P*	0.001	<0.001	<0.001	0.011	<0.001
VMO	*R*	0.376	0.437	0.518	0.237	0.455
	*P*	0.022	0.007	0.001	0.158	0.005
TO	*R*	0.118	0.480	0.266	0.325	0.306
	*P*	0.488	0.001	0.111	0.050	0.065
Attention	*R*	−0.011	0.350	0.139	0.266	0.174
	*P*	0.947	0.033	0.411	0.112	0.302
Total score	*R*	0.553	0.717	0.658	0.561	0.710
	*P*	<0.001	<0.001	<0.001	<0.001	<0.001

*r, correlation coefficient; adjusted P = 0.00125. VP, visual perception; SP, spatial perception; MP, motor praxis; VMO, visuomotor organization; TO, thinking operations; AQ, aphasia quotient; PSA, post-stroke aphasia; WAB, Western Aphasia Battery; LOTCA, Loewenstein Occupational Therapy Cognitive Assessment*.

## Discussion

The present study showed that PSA patients suffered from obvious non-linguistic cognitive impairment. In line with our study, Fonseca et al. (40) have analyzed 47 studies and found that 61.3% of the PSA patients (of a total of 1,710 patients) also had non-linguistic cognitive impairment. Compared to stroke patients without aphasia and healthy individuals, patients with PSA had severe and extensive impairments in multiple cognitive domains, such as orientation, visuospatial perception, VMO, thinking, and attention. This result is similar to that of previous studies (2, 7). This study also showed that non-linguistic cognitive impairment in PSA patients correlated with language impairment. The total LOTCA scores positively correlated with the subscores of WAB and AQ. The AQ scores also positively correlated with some subscores of LOTCA, thus indicating that the severity of the language impairments directly impacts on the severity of the non-linguistic cognition impairments. Other studies have also found that non-linguistic cognitive function could be severely impaired when the severity of aphasia increases (7, 41). Both neuropsychological and neuroimaging studies have provided some evidence on how language and non-linguistic cognition could be closely linked together. The poor performance of non-linguistic cognition in PSA patients can be attributed to the involvement of language in the tasks of attention, executive control, visuospatial perception, and logical thinking (2, 3, 6). Another reason could be due to the disturbance of cognitive networks caused by the impairment of the language network (6).

The VP test examines visual recognition and visual reasoning about objects and shapes. The SP test examines whether patients have spatial agnosia. During the test, except for verbal instruction, we can avoid the impact of comprehension and naming deficits of aphasia on the visuospatial performance by matching the pictures with the target card. In our study, we found the VP score of the PSA group was lower than the non-PSA group and positively correlated with the AQ. Thus, the impairment of VP negatively impacts on the general language function. The present study also observed that VP correlated with repetition, and SP significantly correlated with spontaneous speech, comprehension, and naming. This results are consistent with other studies that confirmed the association between visuospatial dysfunctions and comprehensive or expressive difficulties (10, 15). Encoding of stored speech is one of the critical steps when performing comprehension and production tasks, as well as visuospatial tasks. However, some controversies still exist about how language stimulation and visual image stimulation are stored in the mental lexicon. Gainotti et al. (42) agreed with the hypothesis that speech encoding was separate from image encoding. A study also reported that speech encoding was used in the storage of language and visual information. It was deduced from these studies that one of the mechanisms of repetition was the transmission of information from the visuospatial storage to the language buffer (43). Therefore, the mechanism of language processing such as comprehension, repetition, and production may be similar to the mechanism of visuospatial function.

The VMO test is a result of integrating perceptual activities and movements. In the present study, we also observed that the VMO of PSA patients was significantly impaired, which was related to the impairment of repetition. Although VMO cannot systematically evaluate the executive function, the poor performance may reflect the executive dysfunction to some extent. Previous studies have found that executive dysfunction in aphasia patients can predict the language performance (23, 44, 45), which may be explained by the following deductions. As an important part of executive function, working memory is responsible for short-term storage and manipulation of information needed to perform cognitive tasks, which is also crucial to the complex language processing (43), and this may explain the repetition disorder. Thus, if the executive control system is damaged, the processing of the language cannot be completed. Some researchers have considered that executive dysfunction in PSA may be caused by the comprehension impairment (5). However, in the study, the performance of VMO was not found to be related to the comprehension score. Some studies have also shown that it could not be thoroughly explained by comprehension difficulty (27).

Apart from the VMO test, the MP test can also help determine whether the patient has apraxia using password imitation and physical operation. In our study, it was observed that MP significantly correlated with almost all the language functions with the exception of naming. Apraxia often co-occurs with aphasia, but whether they have common mechanisms or anatomical structures is still not clear (46). It is important to screen for apraxia in PSA patients and to investigate the association between apraxia and aphasia with the use of functional MRI and other neuropsychological methods.

The TO test examines the ability of thinking conversion, judgment, summarization, and reasoning about concepts by using picture-based reasoning and classification tasks. It has been previously reported that patients with aphasia have impairment in logical thinking (15, 47). In this study, the severity of TO positively correlated with the severity of auditory comprehension in patients. This finding suggests that language may play an important role in advanced cognitive activities, and we hypothesized that the impairments of critical language functions, such as comprehension, can affect complex cognitive functions in PSA patients. Logical thinking and reasoning involve many core functions, especially the working memory, which can provide cognitive flexibility for reasoning and solving problems (47). Another possible explanation is the damage to “inner language,” which means patients have difficulty both in comprehension and meaningful verbal output (47).

In the present study, patients presented with attention deficit problems, but no significant association was found between attention and language performance. Previous studies have shown that misdistribution and attention deficits in aphasia patients can lead to difficulty in the production and comprehension of language (12, 13, 48). The training of attention can significantly improve comprehensive performance in aphasia patients (9). Attention is a multidimensional cognitive system, including multiple modules such as vigilance, sustained attention, selective attention, attention switching, and distraction attention (12, 49), which is important for the input and output of language (50). Attention deficit may result in the lack or improper allocation of information resources (28). The potential reason for having negative results on attention in our study may be that the scoring method used for attention is simple and subjective. This also suggests that other attention tests are needed when assessing non-linguistic cognitive functions by LOTCA for PSA.

Upon further analysis, we also found that the total LOTCA scores and the orientation, VP, SP, and MP scores in non-FAG were significantly lower than that in the FAG group. This indicates that patients with non-fluent aphasia have concurrent serious cognitive impairments compared with fluent aphasia patients. This finding is in accordance with the study of Ekaterina et al. (51), in which they evaluated the cognition of stroke patients with fluent aphasia and non-fluent aphasia and found that both groups had non-linguistic cognitive impairment; however, the impaired cognitive domains were more extensive in non-fluent aphasia patients. Another study has also reported that non-fluent aphasia patients were more susceptible to cognitive impairment than fluent aphasia patients (40). This phenomenon may be caused by the difference of lesion location. The stroke lesion of patients with fluent aphasia is often located in the back of brain, and the lesion of patients with non-fluent aphasia is often located in the front of brain (40). The location and size of lesion are important factors of aphasia. In the study, we calculated the lesion volume by the routine MRI/CT scan and found the differences in LOTCA scores between fluent and non-fluent aphasia were independent of lesion volume. Non-fluent aphasia patients had higher NIHSS scores and were more likely to have anterior brain lesions, close to the motor cortex. We supposed the hemiplegia might be one of the reasons for different performance between fluent and non-fluent aphasia, but the patients have been trained to use their contralateral limb to finish tasks. Up to now, there are still not enough studies to provide evidence for the mechanism by which speech fluency and non-linguistic cognition are associated. More attention should be focused on the non-linguistic cognitive assessment of non-fluent aphasia patients in clinical practice.

There are some limitations to the present study. First, the LOTCA is timesaving and easy to master; however, it focuses on the evaluation of perception, visual motor organization, and TOs and ignores the detailed examination of memory, attention, and executive function. The scoring method of attention is subjective, and the severity of cognitive impairment cannot be classified. Therefore, it is necessary to add other domain-specific cognitive tests to assess these cognitive domains and the severity of cognitive impairment. Second, this study focused on the behavioral changes of PSA patients, and functional and structural MRI should be used to explore the mechanism of language and cognitive impairments in future studies. Third, the present study is cross-sectional; hence, we were unable to determine the causal relationship between language and non-linguistic cognitive impairments, as well as to learn about the influence of non-linguistic cognitive training. Longitudinal studies are needed to observe the dynamic changes in these factors to obtain more accurate conclusions. Fourth, the limited sample size of this study might have led to a certain degree of bias in the results.

## Conclusion

In summary, PSA patients have more extensive and serious non-linguistic cognitive impairments compared with stroke patients without aphasia. Patients with non-fluent aphasia often present with serious cognitive impairments than those with fluent aphasia. Non-linguistic cognitive impairments correlate with language impairments in aphasia. These findings need to be validated in large-scale, longitudinal studies.

## Data Availability Statement

All datasets generated for this study are included in the article.

## Ethics Statement

The studies involving human participants were reviewed and approved by the Ethics Committees of Beijing Tiantan Hospital, Capital Medical University, China (ethical approval number: KYSB2016-023). The patients/participants provided their written informed consent to participate in this study.

## Author Contributions

YZhan and GL conceived the study, participated in its design and coordination. JY and XL performed the research and drafted the manuscript. QL, NY, XL, and YZhao evaluated the language and cognitive function. YW, JW, and HC interpreted the lesion information, and extracted the lesion volume. JY and MY performed the statistical analyses. ZH participated in the evaluation of cognition and helped revised the manuscript. All authors have read and approved the final manuscript. All authors contributed to the article and approved the submitted version.

## Conflict of Interest

The authors declare that the research was conducted in the absence of any commercial or financial relationships that could be construed as a potential conflict of interest.
