# Supplementary material for: Characteristics of Non-linguistic Cognitive Impairment in Post-stroke Aphasia Patients
Source: Front Neurol. 2020 Sep 30;11:1038. doi: 10.3389/fneur.2020.01038 (PMC7561418; doi:10.3389/fneur.2020.01038)
Supplement: Supplementary file 1 [file Table_1.pdf]

Appendix-1

Stroke Aphasic Depression Questionnaire in Hospital-10th version(SADQ-H10)

INSTRUCTION: Please indicate on how many days out of the last 7 the patient has shown the following behaviours:

**1. Did he / she have weeping spells?**		**6. Did he / she get angry?**	
no	score 0	no	score 0
1 to 3 days	score 1	1 to 3 days	score 1
4 to 6 days	score 2	4 to 6 days	score 2
every day	score 3	every day	score 3
**2. Did he / she often have restless or have difficulty in falling asleep at night?**		**7. Was he / she unwilling to participate in social activities?**	
no	score 0	no	score 0
1 to 3 days	score 1	1 to 3 days	score 1
4 to 6 days	score 2	4 to 6 days	score 2
every day	score 3	every day	score 3
**3.Did he / she avoid eye contact when you spoke to him / her?**		**8. Did he / she sit without doing anything?**	
no	score 0	no	score 0
1 to 3 days	score 1	1 to 3 days	score 1
4 to 6 days	score 2	4 to 6 days	score 2
every day	score 3	every day	score 3
**4.Did he / she burst into tears or lose control suddenly?**		**9. Did he / she keep himself / herself occupied during the day?**	
no	score 0	no	score 0

1 to 3 days	score 1	1 to 3 days	score 1
4 to 6 days	score 2	4 to 6 days	score 2
every day	score 3	every day	score 3
**5. Did he/she indicate suffering from aches and pains?**		**10. Did he / she get restless and fidgety?**	
no	score 0	no	score 0
1 to 3 days	score 1	1 to 3 days	score 1
4 to 6 days	score 2	4 to 6 days	score 2
every day	score 3	every day	score 3

Total score: _______
