# Supplementary material for: Characteristics of Non-linguistic Cognitive Impairment in Post-stroke Aphasia Patients
Source: Front Neurol. 2020 Sep 30;11:1038. doi: 10.3389/fneur.2020.01038 (PMC7561418; doi:10.3389/fneur.2020.01038)
Supplement: Supplementary file 2 [file Table_2.pdf]

Appendix-2

Loewenstein Occupational Therapy Cognitive Assessment

(LOTCA)

DOMAINS	ITEMS	SCORE RANGE	SCORES
Orientation	1. orientation to place	1 to 8	
	2. orientation to time	1 to 8	
Visual Perception (VP)	3.object identification	1 to 4	
	4. shape identification	1 to 4	
	5. overlapping figures	1 to 4	
	6. object constancy	1 to 4	
Spatial Perception (SP)	7. directions on client's body	1 to 4	
	8. spatial relations	1 to 4	
	9. spatial relations in pictures	1 to 4	
Motor Praxis (MP)	10. motor imitation	1 to 4	
	11. utilization of objects	1 to 4	
	12. symbolic actions	1 to 4	
Visuo-motor Organization (VMO)	13. copying geometric forms	1 to 4	
	14. reproduction of two-dimensional models	1 to 4	
	15. pegboard construction	1 to 4	
	16. colored block designs	1 to 4	
	17. plain block designs	1 to 4	
	18. reproduction of a puzzle	1 to 4	
	19. drawing a clock	1 to 4	

Thinking Operations (TO)	20. pictorial classification	1 to 5	
	21. Riska unstructured object classification	1 to 5	
	22. Riska structured object classification	1 to 5	
	23. pictorial sequencing A	1 to 4	
	24. pictorial sequencing B	1 to 4	
	25. geometrical sequencing	1 to 4	
	26. logical questions	1 to 4	
Attention and Concentration		1 to 4	

Total score: _______
