# Supplementary material for: Characteristics of Non-linguistic Cognitive Impairment in Post-stroke Aphasia Patients
Source: Front Neurol. 2020 Sep 30;11:1038. doi: 10.3389/fneur.2020.01038 (PMC7561418; doi:10.3389/fneur.2020.01038)
Supplement: Supplementary file 3 [file Table_3.pdf]

Appendix-3

Chinese Aphasia Fluency Characteristic Scale

**Spoken Feathers**	**score 1**	**score 2**	**score 3**
**vocabulary**	< 50 characters/minute	51-99 characters/minute	> 100 characters/minute
**intonation**	abnormal	partially normal	normal
**pronunciation**	abnormal	partially normal	normal
**length of phrase**	short, 1-2 characters, telegraphic	partially normal	normal, >4 characters/ sentence
**laboursome speech**	obviously laborious	moderately laborious	effortless
**press of speech**	no	have a tendency	obvious
**substantive words**	rich	rare	lack
**grammar**	no	partially exist	exist
**paraphasia**	no	occasionally	frequently

Total score: _______

Fluent aphasia, from 21 to 27; Intermediate aphasia, from 14 to 20; Non-fluent aphasia, from 9 to 13.
